# Association of *transforming growth factor-β1* gene variants with risk of coal workers' pneumoconiosis^☆^

**DOI:** 10.1016/S1674-8301(10)60038-3

**Published:** 2010-07

**Authors:** Haiyang Qian, Zhifang Song, Meilin Wang, Xiaomin Jia, Aiping Li, Ye Yang, Lianlian Shen, Shasha Wang, Chunhui Ni, Jianwei Zhou

**Affiliations:** a Department of Occupational Medicine and Environmental Health, School of Public Health, Nanjing Medical University, Nanjing 210029, Jiangsu Province, China; b Department of Occupational Health, General Hospital of Xuzhou Mining Business Group Co., Ltd. Xuzhou 221006, Jiangsu Province, China

**Keywords:** coal worker pneumoconiosis, molecular epidemiology, polymorphism, transforming growth factor-β1

## Abstract

**Objective:**

The aim of this case-control study was to explore whether five tagging single nucleotide polymorphisms (tSNPs) within the *transforming growth* *factor*-*β1* (*TGF*-*β1*) gene were involved in manifestation of inflammatory and fibrotic processes associated with coal workers' pneumoconiosis (CWP).

**Methods:**

The study included 508 CWP patients and 526 controls who were underground coal miners from Xuzhou Mining Business Group. Five tSNPs were selected from the HapMap and detected by polymerase chain reaction-restriction fragment length polymorphism (PCR-RFLP) method.

**Results:**

The single SNP analysis showed that the genotype frequencies of SNP2 (rs1800470, +869T/C, extron 1) and SNP5 (rs11466345, intron 5) in CWP cases were significantly different from those in controls. Multivariate logistic regression analysis revealed that SNP2 (rs1800470) CC genotype was associated with decreased risk of CWP (OR = 0.50, 95% CI = 0.32-0.78), which was evident among subgroups of those never smoke (OR = 0.40, 95%CI = 0.24-0.66), cases with stage II (OR = 0.41, 95%CI = 0.22-0.76) and exposure period (< 28 y: OR = 0.54, 95%CI = 0.31-0.95; ≥28 y: OR = 0.52, 95%CI = 0.32-0.96). However, the SNP5 (rs11466345) GG genotype was associated with an increased risk of CWP (OR = 2.5, 95%CI = 1.36-4.57), and further stratification analysis showed that the risk of CWP was increased in both smoking and nonsmoking groups, shorter and longer exposure groups, while the risk of CWP was only increased in patients with stage I and II.

**Conclusion:**

This study suggests that *TGF*-*β1* polymorphisms may contribute to susceptibility of CWP.

## INTRODUCTION

Pneumoconiosis is a global occupational health problem, especially in China with an annual incidence of nearly 80% of all occupational diseases. Inhaling respirable coal dust and silica particulates frequently develop coal workers' pneumoconiosis (CWP), a dust-associated pneumoconiosis characterized by chronic pulmonary inflammation and fibrotic nodular lesions that usually lead to progressive fibrosis[1]. Epidemiological and animal studies have found that silicosis may develop even when exposure was terminated prior to its initial development[2]. Although individuals may be exposed to similar levels of coal dust, only some of them develop lung fibrosis, suggesting that genetic predisposition may influence individual susceptibility to the development of lung fibrosis[3],[4]. Therefore, understanding genetic variability, and the interaction between genetic variation and dust exposure may aid in the identification of high-risk individuals and prevent them from developing CWP.

*Transforming growth factor*-*β1* (*TGF*-*β1*) is an important multifunctional cytokine that modulates myriad cellular and tissue processes, including cell growth, differentiation, apoptosis, and inflammation and is involved in the pathogenesis of the lung fibrosis[5],[6]. The profibrotic effects of *TGF*-*β1* are numerous, including induction of myofibroblasts, increase of matrix synthesis, and inhibition of collagen breakdown. During lung fibrosis, *TGF*-*β1* contributes to the influx and activation of inflammatory cells, transdifferentiation of epithelium to mesenchyme, and influx of fibroblasts and their subsequent elaboration of extracellular matrix[7],[8].

Recently, studies also showed the contribution of *TGF*-*β1* genetic variation to individual fibrosis susceptibility. Several polymorphic variants in the *TGF*-*β1* gene were examined in pulmonary diseases, such as chronic obstructive pulmonary disease (COPD)[9], asthma[10], idiopathic pulmonary fibrosis (IPF)[11], and cystic fibrosis (CF)[12]. Further, most investigators reported a positive correlation between *TGF*-*β1* gene variants and risk of development of these diseases.

Since many single nucleotide polymorphisms (SNPs) are in linkage disequilibrium with other nearby SNPs in the genome, it is feasible to select a small number of SNPs as representative genetic markers, also termed tagging single nucleotide polymorphisms (tSNPs), to capture the common variations in the gene. The use of tSNPs might improve the effectiveness of studies on association, because tSNPs can provide information about nearby SNPs that are not genotyped[13],[14]. However, there are currently only a few reports on the role of *TGF*-*β1* in CWP[15],[16]. In the present study, five tSNPs were selected from data for Chinese subjects in the HapMap (http://www.hapmap.org/) to evaluate the association between genetic variants in *TGF*-*β1* and risk of CWP development in a Chinese population.

## MATERIALS AND METHODS

### Study population

This study consisted of 508 CWP patients and 526 controls, as described previously[17]. Briefly, all subjects were Han Chinese, underground coal miners recruited from coal mines of Xuzhou Mining Business Group Co., Ltd. between January 2006 and December 2008. The high kilovolt chest X-ray and physical examinations were performed for reconfirming the diagnoses based on the China National Diagnostic Criteria for Pneumoconiosis (GBZ 70-2002), which is the same as the 1980 International Labor Office Classification of Pneumoconioses in the judgment of opacity profusion. The controls were matched with CWP cases for age, dust exposure period and job type. Blood sample of 5 ml was obtained from all studied subjects, and used for routine lab tests, including hapetic function tests. Isolated leucocytes were used for DNA extraction. The research protocol was approved by the Institutional Review Board of Nanjing Medical University.

### tSNPs identification and genotyping

**Fig. 1:**
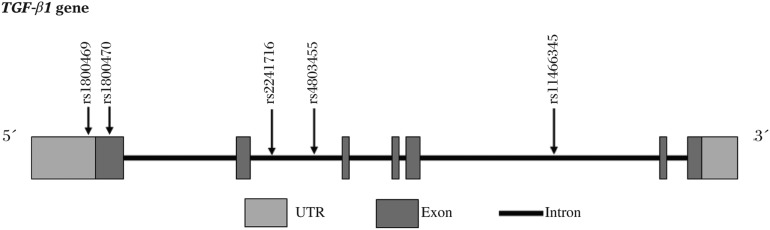
Location of five tSNPs in the *TGF*-*β1* gene.

*TGF*-*β1* is located on chromosome 19q13.1-q13.3 in humans and consists of 7 exons (***Fig. 1***). To examine the gene extensively, this *TGF*-*β1* gene was searched by including its flanking regions of 1,000-bp both upstream and downstream (from 23103114 to 23139491) and a total of 53 SNPs located, including 20 common SNPs [i.e., minor allele frequency (MAF) ≥ 0.05] among a Chinese population included in the HapMap database (HapMap Data Rel 21a/Phase II, Jan07, on NCBI B35 assembly, dbSNP b125). For the genotyping, a set of tSNPs was selected in the *TGFβ1* gene with the following criterion: A minimal set of haplotypes that ensured an R^2^h of at least 0.8 to cover all possible haplotypes that had a frequency of at least 5% as evaluated by the tSNPs program[13]. The reported SNPs in coding region (i.e., rs20577 Thr33Ile in exon1, rs6557634 His141Ar g in exon3, rs20575 Thr209Arg in exon4, rs20576 Glu228Ala in exon5) were not chosen in this study because of their low MAF (< 0.05) in Asian populations. As a result, five tSNPs, which could accurately predict the common (>0.05) haplotypes with a minimum R^2^h of 0.817, were selected. The selected SNP IDs, locations and allele frequencies are summarized in ***Table 1***.

**Table 1:** Primary information on five tSNPs of the *TGF*-*β1* gene among the CWP cases and controls

No.	SNP ID	Base Change	Position^a^	Location	MAF(%)		*P-value^b^*	*P -value* for HWE^C^
					Case	Control		
1	rs1800469	C > T	14128514	Promoter	0.49	0.48	0.581	< 0.001
2	rsl800470	T > C	14127139	Exon 1	0.43	0.48	0.018	< 0.001
3	rs2241716	G > A	14122304	Intron 2	0.36	0.36	0.927	0.059
4	rs4803455	C > A	14119727	Intron 2	0.05	0.05	0.494	0.252
5	rs11466345	A > G	14111679	Intron 5	0.33	0.28	0.015	0.044

^a^ SNP position in NCBI dbSNP; ^b^*P* value for allele distribution difference between case and control; ^c^HWE (Hardy-Weinberg equilibrium) *P* value in the control group.

The selected SNPs were genotyped in all 1,034 subjects by the polymerase chain reaction-restriction fragment length polymorphism (PCR-RFLP) method. The tSNPs information, primers and restriction enzymes of polymorphisms are all listed in ***Table 2***. The polymorphism analysis was performed independently by two individuals in a blinded fashion. About 1% of PCR products were randomly selected and confirmed by sequencing (data not shown), and more than 10% of the samples were randomly selected for repeated genotyping. The results were 100% concordant.

**Table 2:** The tSNP information, primer sequences, annealing temperatures and restriction enzymes for genotyping of *TGF*-*β1* polymorphisms

No.	SNP ID^a^	Base change	Primer	Annealing temperature (°C)	Restriction enzyme
1	rs1800469	C > T	F: 5′AAGGCATGGCACCGCTTCTG3′ R: 5′GAAGGAGGGTCTGTCAACAT3′	57.0	*Bsu36*I
2	rs1800470	T > C	F: 5′CTCCGGGCTGCGGCTGCAGC3′ R: 5′GGCCTCGATGCGCTTCCGCTTCA3′	62.0	*PVU*II
3	rs2241716	G > A	F: 5′TCCACCTAGATGGTCCCTGCA3′ R: 5′CTCCAGCTTGGCAACAGAGTGA3′	59.5	*SDA*I
4	rs4803455	C > A	F: 5′AACTCCTGACCTCAGGAGATC3′ R: 5′CCCTGAATTCTCAGTAACTTAGAATT3′	57.0	*VSP*I
5	rs11466345	A > G	F: 5′CCAAAGTGCTGGGATTACGCG3′ R: 5′TCAAACCCATGTCCAAGGGTCA3′	61.5	*MIU*I

^a^ National Center for Biotechnology Information Reference SNP ID

### Interviewer-administered questionnaire

Information for each person was obtained from the questionnaire, including personal characteristics (age, height, weight, education and family history), occupational history (work type, dust exposure period and personnel protection), occupational diseases (CWP stage, the date of first diagnoses, progress and hearing loss) and life-style (alcohol intake and tobacco smoking). Trained interviewers administered the questionnaires according to standard operating procedures prepared in advance.

### Statistical analysis

Chi-square test was used to evaluate the differences in the frequency distributions of select demographic variables, dust exposure period, smoking status, alleles and genotypes of the *TGF*-*β1* polymorphisms between the CWP cases and controls. Student's *t*-test was used to compare the differences in means of age and dust exposure period between CWP cases and controls. Hardy-Weinberg equilibrium of the controls' genotype distributions was used to test by a goodness-of-fit Chi-square test. Univariate and multivariate logistic regression analysis was performed to obtain crude and adjusted ORs for risk of CWP and their 95% CIs.

## RESULTS

### Characteristics of the study subjects

The selected characteristics of the CWP cases and controls included in the analyses are shown in ***Table 3***. There were no significant differences in the distribution of work types and in the means of exposure period between CWP cases and controls. The average age of CWP cases was older than that of controls (*P* < 0.001). The smoking prevalence of CWP was similar to the controls (*P* = 0.123); however, the pack-years smoked was significantly less than that of controls since the rate of smoking cessation was significantly higher in CWP cases (*P* < 0.001).

**Table 3:** Distribution of selected variables between the CWP cases and controls

Variables	Case (*n* = 508)	Control (*n* = 526)	*P-value*
	*n* (%)	*n* (%)	
Age(y)	69.47 ± 9.70	65.95 ± 6.58	*<* 0.001
Exposure period(y)	26.98 ± 8.68	27.20 ± 7.19	0.664
Work type			
Tunnel/Coal mining	489(96.26)	507(96.39)	0.616
Transport	9(1.77)	12(2.28)	
Others	10(1.97)	7(1.33)	
Smoking status			0.123
Never	289(56.89)	324(61.60)	
Ever	219(43.11)	202(38.40)	
Smoking cessation	111(21.85)	28(5.32)	< 0.001
Pack-years smoked			
0	288(56.69)	322(61.22)	< 0.001
0-20	153(30.12)	77(14.66)	
>20	67(13.19)	127(24.14)	
Stage			
I	237(46.65)		
II	216(42.52)		
III	55(10.83)		

Data are presented as mean±SD or n (%).

### Individual SNP association analysis and risk of CWP

The SNP IDs, locations and allele frequencies are shown in ***Table 1***. SNP1 (rs1800469), SNP2 (rs1800470) and SNP5(rs11466345) of *TGF*-*β1* genotype distributions in the control population deviated significantly from those expected for a population in Hardy-Weinberg equilibrium, while the frequencies of SNP1 and SNP5 genotype in CWP cases were in agreement with Hardy-Weinberg equilibrium (*P* > 0.05). The genotype distributions of the *TGF*-*β1* polymorphisms in cases and controls are summarized in ***Table 4***. The single SNP analysis indicated that the genotype frequencies of SNP2 rs1800470 and SNP5 rs11466345 were significantly different between cases and controls (*P* = 0.003 and *P* = 0.017, respectively). Multivariate logistic regression analysis revealed that the SNP2 (rs1800470) CC genotype was associated with decreased risk of CWP (OR = 0.50, 95% CI = 0.32-0.78) and the SNP5 (rs11466345) GG genotype was associated with an increased risk of CWP (OR = 2.24, 95% CI = 1.37-3.68). No significant association was found between the other SNPs and CWP risk.

**Table 4:** Genotype and frequencies of five tSNPs in *TGF*-*β1* gene among the CWP cases and controls and the associations with risk of CWP

No.	SNP ID	Genotype	Case	Control	*P*	OR (95% CI)	Adjusted OR (95% CI)^a^
			[*n*(%)]	[*n*(%)]			
1	rs1800469	C > T					
		CC	114(22.44)	102(19.39)		1.00	1.00
		TC	273(53.74)	302(57.41)	0.363	0.81(0.59-1.11)	0.86(0.62-1.19)
		TT	121(23.82)	122(23.19)	0.713	0.89(0.62-1.28)	0.93(0.64-1.36)
	*P*		0.402				
2	rs1800470	T > C					
		TT	123(24.21)	109(20.72)		1.00	1.00
		TC	338(66.54)	332(63.12)	0.511	0.90(0.67-1.22)	0.90(0.66-1.23)
		CC	47(9.25)	85(16.16)	0.002	0.49(0.32-0.76)	0.50(0.32-0.78)
	*P*		0.003				
3	rs2241716	G > A					
		GG	219(43.11)	224(42.58)		1.00	1.00
		GA	212(41.73)	223(42.40)	0.868	0.97(0.75-1.27)	0.98(0.75-1.28)
		AA	77(15.16)	79(15.02)	0.507	1.00(0.69-1.44)	0.88(0.61-1.28)
	*P*		0.977				
4	rs4803455	C > A					
		CC	453(89.17)	476(90.49)		1.00	1.00
		CA	55(10.83)	50(9.51)	0.509	1.16(0.77-1.73)	1.15(0.76-1.75)
	*P*		0.482				
5	rs11466345	A > G					
		AA	230(45.28)	266(50.57)		1.00	1.00
		AG	225(43.54)	229(43.54)	0.594	1.14(0.88-1.47)	1.02(0.75-1.40)
		GG	53(10.43)	31(5.89)	0.001	1.98(1.23-3.19)	2.24(1.37-3.68)
	*P*		0.017				

^a^Adjusted for age, smoking and exposure period.

### Stratification analysis between the genotypes of SNPs in *TGF*-*β1* and risk of CWP

As shown in ***Table 5***, the SNP2 (rs1800470) TT genotype was significantly decreased with risk of CWP in subgroups of those never smoke (OR = 0.40, 95%CI = 0.24-0.66), cases with stage II (OR = 0.41, 95%CI = 0.22-0.76) and exposure period (< 28 y: OR = 0.54, 95%CI = 0.31-0.95; ≥28 y: OR=0.52, 95%CI = 0.32-0.96). However, the SNP5 (rs11466345) GG genotype was associated with an increased risk of CWP in both smoking (OR = 0.40, 95%CI = 0.24-0.66) and nonsmoking groups, shorter and longer exposure groups, while the risk of CWP was only increased in patients with stage I and II (***Table 6*** ).

**Table 5:** Stratification analysis between the genotypes of SNP2 rs1800470 polymorphism and CWP risk

Variables	Case/control	Case [n(%)]		Control [n(%)]		*P*	OR (95% CI)	Adjusted OR(95% CI)^a^
		TT/CT	CC	TT/CT	CC			
Total	508/526	461(90.75)	47(9.25)	441(83.84)	85 (16.16)	0.012	0.53(0.36-0.77)	0.54(0.37-0.79)
Exposure period(y)								
<28	221/223	199(90.04)	22(9.96)	185(82.96)	38(17.04)	0.032	0.54(0.31-0.94)	0.54(0.31-0.95)
≥28	287/303	262(91.29)	25(8.71)	256(84.49)	56(15.51)	0.034	0.52(0.31-0.87)	0.56(0.32-0.96)
Smoking status								
Never	289/324	265(91.70)	24(8.30)	264(81.48)	60(18.52)	0.003	0.40(0.24-0.66)	0.40(0.24-0.66)
Ever	219/202	196(89.50)	23(10.50)	177(87.62)	25(12.38)	0.713	0.83(0.46-1.52)	0.89(0.48-1.67)
Stage								
I	237/526	211(89.03)	26(10.97)	441(83.84)	85(16.16)	0.230	0.91(0.56-1.16)	0.80(0.56-1.15)
II	216/526	200(96.59)	16(7.41)	441(83.84)	85(16.16)	0.004	0.42(0.24-0.73)	0.41(0.22-0.76)
III	55/526	50(90.91)	5(9.09)	441(83.84)	85(16.16)	0.172	0.52(0.20-1.34)	0.50(0.18-1.36)

^a^ Adjusted for age, smoking and exposure period.

**Table 6:** Stratification analysis between the genotypes of SNP5 rs11466345 polymorphism and CWP risk

Variables	Case/control	Case [n(%)]		Control [n(%)]		*P*	OR (95% CI)	Adjusted OR(95% CI)^a^
		AA/AG	GG	AA/AG	GG			
Total	508/526	455(89.57)	53(10.43)	495(94.11)	31(5.89)	0.002	1.86(1.17-2.95)	2.17(1.34-3.51)
Exposure period(y)								
<28	221/223	201(90.95)	20(9.05)	213(95.92)	10(4.48)	0.034	2.12(0.97-4.64)	2.31(1.04-5.12)
≥28	287/303	254(88.50)	33(11.50)	282(93.07)	21(6.93)	0.034	1.74(0.98-3.09)	2.08(1.12-3.87)
Smoking status								
Never	289/324	244(84.43)	45(15.57)	310(95.68)	14(4.32)	< 0.001	4.08(2.19-7.61)	4.74(2.49-9.00)
Ever	219/202	211(96.35)	8(3.65)	185(91.58)	17(8.42)	0.049	2.42(1.02-5.74)	2.49(1.00-6.20)
Stage								
I	237/526	209(88.19)	28(11.81)	495(94.11)	31 (5.89)	0.005	2.14(1.25-3.66)	2.18 (1.27-3.74)
II	216/526	193(89.35)	23(10.65)	495(94.11)	31(5.89)	0.019	1.90(1.08-3.35)	2.16 (1.13-4.12)
III	55/526	53(96.36)	2(3.63)	495(94.11)	31(5.89)	0.671	1.66(0.39-7.13)	1.39 (0.31-6.28)

^a^Adjusted for age, smoking and exposure period.

## DISCUSSION

In this case-control study, five tSNPs in the *TGF*-*β1* gene were investigated with respect to an association with the risk of CWP occurrence in Chinese subjects. It was found that the SNP2 (rs1800470) CC genotype decreased the risk of CWP compared to the TT genotype and the further stratification analysis showed that those with the TT genotype had a decreased risk of CWP in subgroups of never smoking, exposure period (< 28 y or ≥28 y) and stage II. However, the SNP5 (rs11466345) GG genotype was associated with an increased risk of CWP compared to the AA genotype; the stratification analysis showed similar trends where GG genotype increased risks of CWP in all subgroups except stage III.

*TGF*-*β1* was found to play a key role in the development of lung fibrosis, and increased expression of *TGF*-*β1* occurred in lung tissue in patients with lung fibrosis and animal models of pulmonary fibrosis[8],[18]–[20]. Yao et al[15] noted the potential role of TGF-β1 genetic variants in the pathogenesis of CWP by demonstrating that the TGF-β1 gene −509 site (rs1800469) polymorphism influenced the concentration of TGF- β1 in serum, but was not different among stages and exposure period of CWP. In contrast, Wu et al[21] did not find an association between the TGF-β1 gene polymorphisms at positions −509 (rs1800469), +869 (rs1800470), and +915 (rs1800471) with silicosis risk in Chinese iron miners.

In the present study, we found that SNP2 (rs1800470, T > C) and SNP5 (rs11466345, A > G) were significantly associated with CWP risk. SNP2 (rs1800470) is an exon polymorphism whose T > C transition is at nucleotide +869, which leads to the Leu10Pro amino acid substitution. Previous studies reported that *TGF*-*β1* gene variants were important genetic modifiers of lung disease progression since the genotypes of +869T/C (rs1800470) can influence *TGF*-*β1* plasma levels[22],[23]. The SNP5 is an intronic polymorphism whose functional consequences are less intuitive, but intronic polymorphisms have been reported to be associated with a variety of chronic diseases including breast cancer[24], essential hypertension[25] and type II diabetes[26]. By analyzing the putative transcription factors for *TGF*-*β1* from the Genomatix program (http://www.genomatix.de), the SNP5 A locus is found to be centered in a 21 bp that is nearly identical to the Tax/CREB complex binding site, and the SNP5 G locus is in 25bp nearly identical to the arylhydrocarbon-receptor nuclear translocator (ARNT). SNP5 (rs11466345) variants may influence the affinities of Tax/CREB complex and/or ARNT with the binding sites. Although exact molecular mechanisms underlying variation in SNP5 and the ability to increase risk of CWP are unknown, it is possible that this SNP may exert an effect on gene expression and be in linkage disequilibrium with other functional variants. However, further investigation is required to confirm these hypotheses.

Studies on the associations between the *TGF*-*β1* gene polymorphisms and susceptibility of lung fibrosis have been reported[11],[12],[27], but with few involved CWP. Yucesoy et al[16] found no association in coal miners between TGF-β1 genetic variations of promoter region (rs1800469) in patients with susceptibility to progressive massive fibrosis. In the present case-control study, 5 tSNPs of TGF-β1 were examined, and data showed that although the result of SNPs in promoter region was similar to the study reported by Yucesoy et al[16], the variants in exon 1 (rs1800470) and intron 5 (rs11466345) were associated with susceptibility to CWP development.

In this study, cases and controls matched very well in work type and exposure period, which were the most important matching indicators. The smoking frequency of cases was not significantly different from controls, but over 50% smoking cases stopped smoking, much higher than that of controls (13.86%), due to the breathing problems. The age was not matched very well and this needs to be improved in later studies. The frequencies of SNP3 and SNP4 genotypes in controls and the frequencies of SNP1 and SNP5 genotypes in cases were in agreement with Hardy-Weinberg equilibrium, which indicated that our controls may differ from the general population since the controls were from underground miners who had the same dust exposure as the cases, but without lung fibrosis. Therefore, a control group from the local general population is needed in a future study.

In conclusion, our study demonstrated that some representative genetic variants in *TGF*-*β1* may exert a role in the risk of CWP development. Studies with ethnically diverse populations and functional evaluation are warranted to confirm our findings.
